# Quorum Sensing Signal Selectivity and the Potential for Interspecies Cross Talk

**DOI:** 10.1128/mBio.00146-19

**Published:** 2019-03-05

**Authors:** Samantha Wellington, E. Peter Greenberg

**Affiliations:** a Department of Microbiology, University of Washington, Seattle, Washington, USA; Cornell University; University of Wisconsin-Madison; International Centre for Genetic Engineering and Biotechnology

**Keywords:** acyl-homoserine lactone, bacterial communication, gene regulation, transcription factors

## Abstract

Many species of proteobacteria communicate with kin and coordinate group behaviors through a form of cell-cell signaling called acyl-homoserine lactone (AHL) quorum sensing (QS). Most AHL receptors are thought to be specific for their cognate signal, ensuring that bacteria cooperate and share resources only with closely related kin cells. Although specificity is considered fundamental to QS, there are reports of “promiscuous” receptors that respond broadly to nonself signals. These promiscuous responses expand the function of QS systems to include interspecies interactions and have been implicated in both interspecies competition and cooperation. Because bacteria are frequently members of polymicrobial communities, AHL cross talk between species could have profound impacts. To better understand the prevalence of QS promiscuity, we measured the activity of seven QS receptors in their native host organisms. To facilitate comparison of our results to previous studies, we also measured receptor activity using heterologous expression in Escherichia coli. We found that the standard E. coli methods consistently overestimate receptor promiscuity and sensitivity and that overexpression of the receptors is sufficient to account for the discrepancy between native and E. coli reporters. Additionally, receptor overexpression resulted in AHL-independent activity in Pseudomonas aeruginosa. Using our activation data, we developed a quantitative score of receptor selectivity. We find that the receptors display a wide range of selectivity and that most receptors respond sensitively and strongly to at least one nonself signal, suggesting a broad potential for cross talk between QS systems.

## Abstract (precis)

Specific recognition of cognate signals is considered fundamental to cell signaling circuits as it creates fidelity in the communication system. In bacterial quorum sensing (QS), receptor specificity ensures that bacteria cooperate only with kin. There are examples, however, of QS receptors that respond promiscuously to multiple signals. “Eavesdropping” by these promiscuous receptors can be beneficial in both interspecies competition and cooperation. Despite their potential significance, we know little about the prevalence of promiscuous QS receptors. Further, many studies rely on methods requiring receptor overexpression, which is known to increase apparent promiscuity. By systematically studying QS receptors in their natural parent strains, we find that the receptors display a wide range of selectivity and that there is potential for significant cross talk between QS systems. Our results provide a basis for hypotheses about the evolution and function of promiscuous signal receptors and for predictions about interspecies interactions in complex microbial communities.

## INTRODUCTION

**FIG 1:**
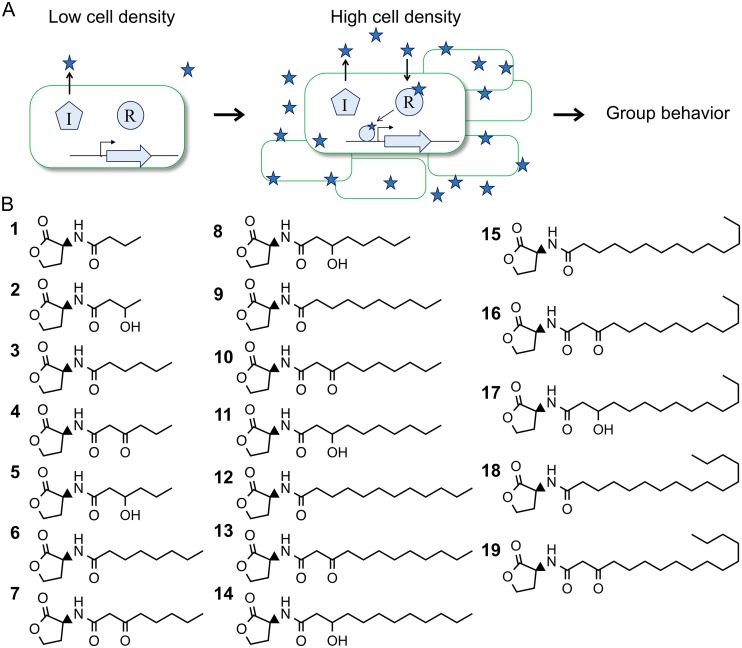
Diagram of a generic AHL QS circuit and structures of AHLs used in this study. (A) AHL QS systems generally contain a synthase (I) that produces an AHL signal, depicted here as a star. The signal acyl chains vary in length from 4 to 20 carbons, with potential hydroxy or oxo modification on the 3rd carbon, double bonds, branching, and/or terminal aryl moieties. At low cell densities, signals diffuse away from cells. At high cell densities, signals accumulate and can bind the QS receptor (R), which is a cytosolic transcription factor that regulates genes involved in group behaviors. (B) Chemical structures of AHLs used. Non-IUPAC descriptions of compounds are as follows: (1) C4-HSL; (2) 3OHC4-HSL; (3) C6-HSL; (4) 3OC6-HSL; (5) 3OHC6-HSL; (6) C8-HSL; (7) 3OC8-HSL; (8) 3OHC8-HSL; (9) C10-HSL; (10) 3OC10-HSL; (11) 3OHC10-HSL; (12) C12-HSL; (13) 3OC12-HSL; (14) 3OHC12-HSL; (15) C14-HSL; (16) 3OC14-HSL; (17) 3OHC14-HSL; (18) C16-HSL; (19) 3OC16-HSL.

Many bacteria use quorum sensing (QS) to communicate with kin and coordinate group behaviors ranging from antibiotic production to virulence factor secretion and biofilm formation (1). In many proteobacteria, QS is mediated by acyl-homoserine lactone (AHL) signals. AHL QS systems consist of a signal synthase and a dimeric cytosolic receptor that serves as a transcriptional activator or repressor (Fig. 1A). AHLs can diffuse through cellular membranes (2, 3) and are comprised of a homoserine lactone core with an acyl tail (Fig. 1B). Most known AHLs possess fatty acyl tails that vary in length from 4 to 20 carbons and in modifications, particularly at the third carbon, which can be unsubstituted or have a hydroxy or oxo modification. To date, roughly 20 different naturally produced fatty AHLs have been identified among hundreds of quorum sensing organisms (4, 5). Thus, there is some degeneracy whereby QS systems from different organisms produce and respond to the same signal. Despite the very similar structures of natural AHL signals, receptors are believed to be highly specific for and sensitive to their cognate signal (6). There are, however, reported exceptions to this paradigm. For example, Chromobacterium violaceum is frequently used as a tool for AHL detection due to its receptor’s promiscuous response to multiple AHLs (7). Furthermore, “eavesdropping” through promiscuous receptors has been shown to affect both interspecies competition (8) and cooperation (9) in laboratory experiments. In *in vivo* settings, interspecies cross talk via degenerate signals and/or promiscuous receptors have both been shown to modulate bacterial virulence to the benefit or detriment of a plant host (9–11), and similar interactions have been hypothesized to occur during human infections (12). Given that most bacteria are found in mixed polymicrobial communities, it is tempting to speculate that cross talk between QS systems mediates numerous interspecies interactions (13, 14).

Despite their potential importance, we know little about the prevalence, function, and evolution of promiscuous QS receptors. There have been prior studies aimed at comprehensively profiling the responses of a set of receptors to large sets of natural and synthetic ligands (15, 16), and multiple studies have measured the selectivity of individual receptors against smaller sets of AHLs (7, 17–23). These studies were limited, however, in that many of them used heterologous expression of the receptors in Escherichia coli. Due to tractability, signal preferences and receptor selectivity are frequently studied using E. coli engineered to report receptor activity (24). Such methods require artificial expression of the AHL receptor, likely to a higher degree than the receptor’s natural expression level. Importantly, increased AHL receptor expression has been linked to increased sensitivity and promiscuity (19, 25), and a previous study comparing LasR receptor activity in its parent species, Pseudomonas aeruginosa, with heterologous expression in E. coli found significant discrepancies between these two methods (26). Previous reports may, therefore, overestimate receptor promiscuity and the potential for cross talk.

We sought to systematically study QS receptor selectivity in the receptors’ natural parent strains, thereby avoiding overexpression and enabling more robust predictions of how bacteria would respond to nonself signals in nature. We selected seven receptors for our characterization: LuxR from Vibrio fischeri, CviR from C. violaceum, LasR, RhlR, and QscR from P. aeruginosa, and BtaR1 and BtaR2 from Burkholderia thailandensis (Table 1). These organisms range from soil saprophytes (C. violaceum and B. thailandensis) to a squid symbiont (V. fischeri) to human (P. aeruginosa and C. violaceum) and plant (P. aeruginosa) pathogens and, with the exception of V. fischeri, are frequently members of polymicrobial communities (27–32). Their receptors control a variety of processes, including antibiotic production (CviR and BtaR2), extracellular enzyme production (LasR, RhlR, and CviR), and luminescence (LuxR). Critically, the selected QS systems are well described, enabling study of their activation in the bacteria that naturally express them.

**TABLE 1:** Sensitivity of AHL receptors to cognate signals

Organism	Receptor	Signal	EC_50_^*a*^	E. coli EC_50_^*b*^
P. aeruginosa	LasR	3OC12-HSL	593 ± 128 nM	12.9 ± 3.6 nM
	RhlR	C4-HSL	>100 µM^*d*^	122 ± 17 µM
	QscR	3OC12-HSL^*c*^	1.90 ± 0.27 µM	53.4 ± 11.3 nM
B. thailandensis	BtaR1	C8-HSL	50.5 ± 4.6 nM	10.5 ± 3.6 nM
	BtaR2	3OHC10-HSL	15.0 ± 5.3 nM	60.6 ± 16.0 nM
V. fischeri	LuxR	3OC6-HSL	272 ± 15 nM	NT
C. violaceum	CviR	C6-HSL	83.4 ± 24.5 nM	NT

a EC_50_ is the concentration required for half-maximal activity of the receptor in its native host.

b EC_50_ values for activation of receptors heterologously expressed in E. coli (DH5α). NT, not tested.

c QscR is an orphan/solo receptor and does not have a paired signal synthase, but it does respond to 3OC12-HSL produced by LasI.

d RhlR activity was not saturated at 1 mM C4-HSL.

To measure selectivity, we quantified receptor responses to a panel of synthetic AHL signals, calculating both percent activation and concentration of half-maximal activation (EC_50_) for each signal. To better compare our results to previous studies, we also made the same measurements using heterologous expression of the receptors in E. coli. The E. coli reporters consistently overestimated sensitivity and promiscuity for our selected receptors. We determined that overexpression of the receptors is sufficient to account for the differences between E. coli and native reporters. Surprisingly, we also found that overexpression of the receptors can lead to AHL-independent activity in P. aeruginosa.

By using our activation data, we developed a quantitative selectivity score for each receptor. We found that the receptors display a wide range of signal preferences and selectivity. Some receptors, such as RhlR, are highly specific for their cognate signal, while others, such as BtaR2, are very promiscuous. The remaining receptors are on a continuum, with many displaying intermediate levels of selectivity and the ability to respond strongly and sensitively to at least one noncognate signal. These results suggest the potential for significant AHL-mediated interspecies interactions in nature and are a prelude to understanding the evolution of signal and receptor diversity.

## RESULTS

### Construction of reporters in native hosts and E. coli.

C. violaceum and V. fischeri each possess an AHL receptor, CviR and LuxR, respectively, that controls a readily measured phenotype, production of the purple antibiotic violacein and luminescence, respectively (7, 17). We used AHL synthase-null mutants of each organism (see Table S1 in the supplemental material), such that only exogenously provided AHLs were present, and measured violacein production or luminescence to quantify QS activation. For both organisms, QS activation required exogenous AHL, and the concentration of half-maximal activation (EC_50_) of their cognate AHLs was in the mid-nanomolar range (Table 1), consistent with previous reports (17, 18).

10.1128/mBio.00146-19.7 TABLE S1 Bacterial strains used in this study. Download Table S1, DOCX file, 0.1 MB. Copyright © 2019 Wellington and Greenberg. 2019 Wellington and Greenberg This content is distributed under the terms of the Creative Commons Attribution 4.0 International license.

P. aeruginosa has two complete AHL QS circuits, the Las and Rhl systems, as well as an orphan/solo receptor, QscR (33, 34). Although QscR does not have its own signal synthase, it responds to the *N-*3-oxo-dodecanoyl-L-homoserine lactone (3OC12-HSL) signal produced by LasI, which is paired to the transcription factor LasR (20). To measure QS activity in P. aeruginosa, we used receptor-responsive promoters to control the expression of *gfp* in an AHL synthase-null mutant (PAO-SC4). We used the well-validated promoters P_rsaL_, P_rhlA_, and P_PA1897_ for LasR, RhlR, and QscR, respectively (20, 35, 36). We compared two methods for measuring LasR activation in P. aeruginosa: plasmid-borne *gfp* controlled by P_rsaL_ and chromosomal integration of the same promoter-*gfp* construct. The two methods produced identical responses to a panel of 19 AHL signals and comparable EC_50_ values for LasR’s cognate AHL, 3OC12-HSL, as well as for two of the most active AHLs from the panel (see Fig. S1 in the supplemental material). On the basis of these results, we opted to use plasmid-based reporters for all further studies. The reporters for all three P. aeruginosa receptors were responsive to their receptor’s cognate signal (Fig. S2A). In our P. aeruginosa strain, PAO1, LasR positively regulates the transcription of *rhlR*, and activation of LasR via exogenous 3OC12-HSL is required for high-level RhlR expression in the synthase-null mutant (37). As expected, in the RhlR reporter strain PAO-SC4 (pPROBE-P_rhlA_), both 3OC12-HSL and the RhlR cognate signal, *N*-butyryl-L-homoserine lactone (C4-HSL), were required for significant RhlR activity (Fig. S2A).

10.1128/mBio.00146-19.1 FIG S1 LasR activity in P. aeruginosa measured via a plasmid-based or chromosomal reporter. (A) Activation of LasR in the P. aeruginosa synthase-null mutant (PAO-SC4) by a panel of AHL signals (100 µM). Activation was measured via a plasmid-based reporter (pPROBE-P_rsaL_; “Plasmid”, gray bars) or via integration of the reporter construct into the chromosome (PAO-SC4-P_rsaL_-*gfp*; “Chromosome”, black bars). Activation is normalized to 100 µM 3OC12-HSL. Three of the most active signals, 3OC12-HSL (B), 3OC14-HSL (C), and C12-HSL (D) were tested in dose-response experiments against the synthase-null mutant with the plasmid-based reporter (strain PAO-SC4 carrying pPROBE-_PrsaL_; black squares) or the chromosomally integrated reporter (PAO-SC4-P_rsaL_-*gfp*; open circles). Data are the means and ranges for two biological replicates and are representative of three independent experiments. Download FIG S1, TIF file, 0.7 MB. Copyright © 2019 Wellington and Greenberg. 2019 Wellington and Greenberg This content is distributed under the terms of the Creative Commons Attribution 4.0 International license.

10.1128/mBio.00146-19.2 FIG S2 Validation of AHL receptor activity reporters. (A) Activity of LasR, QscR, and RhlR measured via pPROBE-P_rsaL_, -P_PA1897_, and -P_rhlA_, respectively, in the synthase-null P. aeruginosa mutant (PAO-SC4) with signal (10 µM) treatment as indicated. Activity was measured as fluorescence (RFU) relative to cell density (OD_600_). (B) Activity of BtaR1 measured via pPROBE-P_cdiA_ in wild-type B. thailandensis (E264; WT), the synthase-null mutant (JBT112; ΔI^3^), or the BtaR1 deletion mutant (JBT107; ΔR1) with C8-HSL (10 µM) treatment as indicated. (C) Activity of BtaR2 measured via pPROBE-P_btaK_ in wild-type B. thailandensis (E264; WT), the synthase-null mutant (JBT112; ΔI^3^), or the BtaR2 deletion mutant (JBT108; ΔR2) with 3OHC10-HSL (10 µM) treatment as indicated. (D to H) Activity of E. coli reporters of AHL receptor activation with arabinose and/or AHL (10 µM) treatment as indicated for LasR [DH5α carrying pJNL and pPROBE-P_rsaL_ (5α (pJNL, pPROBE-P_rsaL_))] (D), QscR (5α (pJNQ, pPROBE-P_PA1897_)) (E), RhlR (5α (pJNR, pPROBE-P_rhlA_)) (F), BtaR1 (5α (pJNR1, pPROBE-P_cdiA_)) (G), or BtaR2 (5α (pJNR2, pPROBE-P_btaK_)) (H). Data are the means and SEM from two (D to H) or three (A to C) independent experiments. Download FIG S2, TIF file, 1.3 MB. Copyright © 2019 Wellington and Greenberg. 2019 Wellington and Greenberg This content is distributed under the terms of the Creative Commons Attribution 4.0 International license.

B. thailandensis has three complete AHL QS circuits (BtaI-R1 to -R3) as well as two orphan/solo receptors, BtaR4 and BtaR5 (32). There is no known phenotype associated with the BtaI-R3 system, BtaR4 (MalR) exerts AHL-independent control of its transcriptional regulon, and BtaR5 has no known regulon (32, 38). Therefore, we focused on BtaR1 and BtaR2 for our studies. BtaR1 controls a contact-dependent type VI secretion toxin-immunity system (32, 39), and BtaR2 controls synthesis of the antibiotic bactobolin (21). We selected the following promoters as reporters of BtaR1 and BtaR2 activity, respectively: P_cdiA_ and P_btaK_. For each reporter, we validated that *gfp* expression requires both AHL and a functional receptor (Fig. S2B and C).

To better compare our results to more standard methods, we also constructed reporters in E. coli. The E. coli reporters contained the same plasmid-borne promoter-*gfp* constructs used in the parent strains along with a plasmid coding for an arabinose-inducible receptor gene. These reporters required both receptor expression and exogenous AHL for fluorescence (Fig. S2D to H). For each P. aeruginosa receptor, the corresponding E. coli reporter overestimated sensitivity to the cognate AHL by at least 10-fold (Table 1). The E. coli BtaR1 reporter also overestimated sensitivity to its cognate AHL by about fivefold (Table 1). BtaR2 is the only exception; the receptor is more sensitive to its cognate signal in B. thailandensis than in the E. coli reporter.

### AHL receptors display a variety of signal preferences.

**FIG 2:**
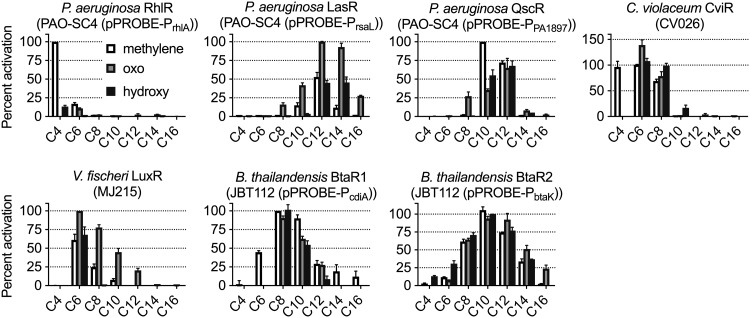
Activation of receptors in native organisms. Synthase-null mutants (PAO-SC4, CV026, MJ215, and JBT112) harboring receptor activity reporters were treated with the indicated AHLs (100 µM), which are labeled by length of acyl chain and modification on the 3rd carbon. The RhlR reporter PAO-SC4 (pPROBE-P_rhlA_) was pretreated with 3OC12-HSL (10 µM). For all reporters, activation is normalized to that of the receptor’s most potent signal (cognate AHL for all receptors except QscR, which is normalized to C10-HSL). *N-*3-oxobutyryl-L-homoserine lactone (3OC4-HSL) and *N-*3-hydroxyhexadecanoyl-L-homoserine lactone (3OHC16-HSL) were not included in the panel. Values are means plus SEM (error bars) from *n* ≥ 3 independent experiments.

To determine signal preferences, we tested a panel of 19 AHLs against each receptor (Fig. 1B and 2 and Fig. S3). The panel contains the majority of the naturally produced fatty AHLs identified thus far (4). AHLs are produced in laboratory cultures at concentrations up to mid-micromolar (40). For these studies, we used 100 µM AHL and then further refined our data through dose-response experiments. Although the majority of receptors responded to roughly half of the signals in the panel, RhlR stands out in that it responded only very weakly to noncognate signals. On the other end of the spectrum, BtaR2 appears to be highly promiscuous, responding to all but two of the signals in the panel.

10.1128/mBio.00146-19.3 FIG S3 Activation of AHL receptors expressed in E. coli. E. coli (5α) reporter strains harboring an AHL receptor gene on a plasmid (pJNR, pJNL, pJNQ, pJNR1, or pJNR2 for *rhlR*, *lasR*, *qscR*, *btaR1*, and *btaR2*, respectively) and an activity reporter plasmid (pPROBE-P_rhlA_, -P_rsaL_, -P_PA1897_, -P_cdiA_, or -P_btaK_ for RhlR, LasR, QscR, BtaR1 and BtaR2, respectively) were treated with the indicated AHLs (100 µM). Activation is normalized to that of the receptor’s most potent signal (cognate AHL for all receptors except QscR, which is normalized to C10-HSL). AHLs are labeled by length of acyl chain and modification on the 3rd carbon. *N-*3-oxobutyryl-L-homoserine lactone (3OC4-HSL) and *N-*3-hydroxyhexadecanoyl-L-homoserine lactone (3OHC16-HSL) were not included in the panel. Bars show the means and SEMs from *n* ≥ 3 independent experiments. Download FIG S3, TIF file, 1.1 MB. Copyright © 2019 Wellington and Greenberg. 2019 Wellington and Greenberg This content is distributed under the terms of the Creative Commons Attribution 4.0 International license.

In general, the receptors were preferentially activated by AHLs with either a particular substituent and/or a certain length of acyl chain. LuxR and LasR displayed a strong preference for oxo-substituted AHLs. BtaR1 responded most strongly to AHLs with unsubstituted acyl chains, and BtaR2 showed a preference for 10-carbon AHLs. Consistent with previous reports (7, 18), CviR was strongly activated by AHLs with short acyl chains. Interestingly, QscR does not have as clear a preference for acyl chain length or substituents as do the other receptors. Consistent with previous findings using E. coli reporters (20, 41–43), QscR responded strongly to nonself signals. While QscR was most responsive to *N*-decanoyl-L-homoserine lactone (C10-HSL), the next most active signals each have a 12-carbon acyl chain.

For most of the receptors, the E. coli reporters responded to a larger number of signals than the native reporters (Fig. S3). They also displayed a larger degree of activation by noncognate signals, such that many AHLs activated to the same degree as the receptor’s cognate signal. BtaR2 is an exception. Although most noncognate AHLs were more active against BtaR2 in the E. coli reporter, a few were less active, suggesting the potential for more complex regulation of BtaR2 activity or of *btaK* transcription in B. thailandensis.

Although our study is focused on the activation of AHL receptors, noncognate AHLs can also inhibit receptor activity through a variety of mechanisms (7, 15, 16, 18, 41–44). To explore inhibitory effects of noncognate AHLs, we tested our AHL panel for inhibition of LasR, QscR, CviR, or LuxR activity. As with activation, the receptors displayed a range of sensitivities to inhibition. Although LuxR and CviR were strongly inhibited by multiple AHLs, QscR displayed an intermediate level of susceptibility, and LasR was largely insensitive to inhibition (Fig. S4A to D). We considered the possibility that the differences in sensitivity to inhibition could be due, in part, to differences in the receptors’ EC_50_ for their cognate AHLs. To study inhibition, we treated each reporter strain with the EC_50_ of its cognate AHL and then with 100 µM of each AHL in the panel. Because CviR and LuxR each have a lower EC_50_ than LasR or QscR, the ratio of inhibitor to cognate AHL was greater in studies of these two receptors. To address this concern, we tested the AHL panel at 10× the EC_50_ of each cognate AHL ([AHL panel] = 800 nM for CviR and 2.7 µM for LuxR). Even at this lower concentration, LuxR and CviR were strongly inhibited by several AHLs in the panel (Fig. S4E and F).

10.1128/mBio.00146-19.4 FIG S4 Inhibition of AHL receptors in their native backgrounds. AHL receptor reporter strains for LasR (PAO-SC4 (pPROBE-P_rsaL_)), CviR (CV026), LuxR (MJ215), or QscR (PAO-SC4 (pPROBE-P_PA1897_)) were treated with their cognate AHL (3OC12-HSL for LasR and QscR, C6-HSL for CviR, and 3OC6-HSL for LuxR; concentration of each cognate AHL was equal to EC_50_). Strains were then treated with each indicated AHL (100 µM in panels A to D; 800 nM in panel E; 2.7 µM in panel F), and receptor activity was measured via fluorescence, luminescence, or violacein in order the quantify the inhibitory effect of each AHL. Bars show the means and SEM of *n* ≥ 2 independent experiments. Download FIG S4, TIF file, 1.2 MB. Copyright © 2019 Wellington and Greenberg. 2019 Wellington and Greenberg This content is distributed under the terms of the Creative Commons Attribution 4.0 International license.

### Development of a quantitative selectivity score.

**FIG 3:**
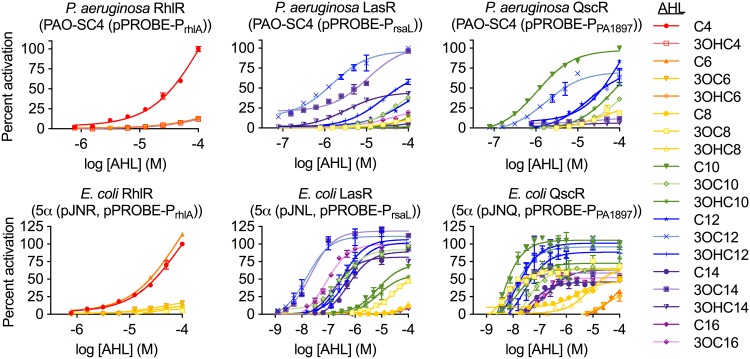
Dose-response curves showing activation of AHL receptors in P. aeruginosa or in recombinant E. coli. Activation was measured via promoter-*gfp* reporter constructs (pPROBE-P_rhlA_, -P_rsaL_, and -P_PA1897_, for RhlR, LasR, and QscR, respectively) in the synthase-null P. aeruginosa mutant (PAO-SC4) or in E. coli (DH5α) harboring the indicated receptor gene on a plasmid (pJNR, pJNL, and pJNQ for *rhlR*, *lasR*, and *qscR*, respectively). The P. aeruginosa RhlR reporter PAO-SC4 (pPROBE-P_rhlA_) was pretreated with 3OC12-HSL (10 µM). Activation is normalized to that of the receptor’s most potent signal. Data show the means and ranges for two biological replicates and are representative of *n* ≥ 3 independent experiments. See Tables S3 and S4 in the supplemental material for EC_50_ values.

To better quantify signal preferences and receptor selectivity, we tested each active signal in a dose-response format. The three P. aeruginosa receptors illustrate the range of receptor selectivity observed. RhlR is very specific for its cognate signal (C4-HSL) (Fig. 3 and Table S3). Interestingly, unlike other AHL receptors which are saturated at relatively low concentrations of their cognate signal, RhlR activity was not saturated even at 1 mM C4-HSL (Fig. S5). LasR is also fairly selective, responding sensitively to its cognate signal (3OC12-HSL) and to only one other AHL, *N-*3-oxotetradecanoyl-L-homoserine lactone (3OC14-HSL) (Fig. 3). QscR is more promiscuous than LasR and is, in fact, most sensitive to C10-HSL, a signal that is not produced by P. aeruginosa. In all cases, the corresponding E. coli reporter overestimated the receptor’s sensitivity to both cognate and noncognate signals (Fig. 3 and Table S4). These trends hold true for the B. thailandensis receptors as well (Fig. S5). Notably, BtaR1 is about 15-fold and BtaR2 is about 5-fold more sensitive to the BtaI-R3 signal, *N*-3-hydroxyoctanoyl-L-homoserine lactone (3OHC8-HSL), in the E. coli reporters than in the native B. thailandensis reporters. E. coli reporter methods may, therefore, overestimate not only the potential for interspecies cross talk but also for cross talk between QS systems within a single organism.

10.1128/mBio.00146-19.5 FIG S5 Dose-response curves showing activation of AHL receptors in native organisms or in E. coli. Activation of P. aeruginosa and B. thailandensis receptors was measured via promoter-*gfp* reporter plasmids (pPROBE-P_rhlA_, -P_cdiA_, and -P_btaK_, for RhlR, BtaR1, and BtaR2, respectively) in the synthase-null P. aeruginosa mutant (PAO-SC4) or B. thailandensis mutant (JBT112) or in E. coli (5α) harboring the indicated receptor gene on a plasmid (pJNR, pJNR1, and pJNR2 for *rhlR*, *btaR1*, and *btaR2*, respectively). Activation of LuxR and CviR was measured in synthase-null V. fischeri (MJ215) or C. violaceum (CV026), respectively. The P. aeruginosa RhlR reporter PAO-SC4 (pPROBE-P_rhlA_) was pretreated with 3OC12-HSL (10 µM). Activation is normalized to that of the receptor’s most potent signal. Data show the means and ranges for two biological replicates and are representative of *n* ≥ 3 independent experiments. See Tables S3 and S4 for EC_50_ values. Download FIG S5, TIF file, 2.1 MB. Copyright © 2019 Wellington and Greenberg. 2019 Wellington and Greenberg This content is distributed under the terms of the Creative Commons Attribution 4.0 International license.

10.1128/mBio.00146-19.8 TABLE S2 Plasmids used in this study. Download Table S2, DOCX file, 0.1 MB. Copyright © 2019 Wellington and Greenberg. 2019 Wellington and Greenberg This content is distributed under the terms of the Creative Commons Attribution 4.0 International license.

10.1128/mBio.00146-19.9 TABLE S3 EC_50_ values for receptors in their native hosts. The EC_50_ values are shown in micromolar unless indicated otherwise. Download Table S3, DOCX file, 0.1 MB. Copyright © 2019 Wellington and Greenberg. 2019 Wellington and Greenberg This content is distributed under the terms of the Creative Commons Attribution 4.0 International license.

10.1128/mBio.00146-19.10 TABLE S4 EC_50_ values for each indicated AHL for receptors expressed in E. coli. The EC_50_ values are shown in micromolar unless indicated otherwise. Download Table S4, DOCX file, 0.1 MB. Copyright © 2019 Wellington and Greenberg. 2019 Wellington and Greenberg This content is distributed under the terms of the Creative Commons Attribution 4.0 International license.

To date, classification of QS receptors as “specific” or “promiscuous” has largely been qualitative. Semiquantitative scores have been used to make pairwise comparisons of relative selectivity between receptors (43) or signals (25), but there has yet to be a single score that represents a receptor’s response to every signal. We sought to develop a quantitative score of selectivity such that the receptors could be more robustly compared to one another. We began by calculating the area under the curve (AUC) for the dose-response curves of each receptor-signal pair. AUC takes into account both sensitivity (EC_50_) and degree of activation. It is, therefore, a robust measurement of global activity and has been used to rank compounds and to determine selectivity in fields such as cancer drug discovery (45). To quantify receptor selectivity, we divided the AUC of the receptor’s response to its most potent AHL by the sum of its responses to all other AHLs. Using this formula, the more selective the receptor, the higher the score. Based on this score, RhlR is the most specific receptor and BtaR2 is the most promiscuous (Table 2). The remaining receptors are on a continuous spectrum of intermediate selectivity (RhlR > LuxR > LasR > BtaR1 > QscR > CviR > BtaR2). E. coli reporters roughly maintain the order of receptor selectivity while strongly overestimating promiscuity for all receptors.

**TABLE 2:** Selectivity scores for AHL receptors in native organisms or in E. coli

Receptor	Native selectivity score	E. coli selectivity score^*a*^
RhlR	1.80 ± 0.09	0.70 ± 0.13
LuxR	0.97 ± 0.16	NT
LasR	0.62 ± 0.09	0.20 ± 0.02
BtaR1	0.58 ± 0.06	0.26 ± 0.03
QscR	0.52 ± 0.06	0.14 ± 0.03
CviR	0.39 ± 0.06	NT
BtaR2	0.22 ± 0.03	0.17 ± 0.03

a NT, not tested.

### Overexpression of receptors is sufficient to increase sensitivity and promiscuity.

**FIG 4:**
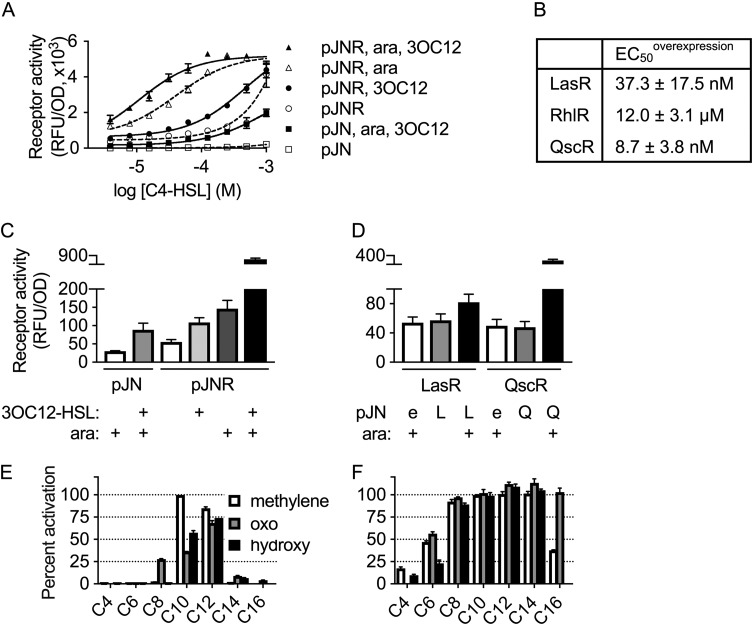
Effect of overexpression on AHL receptor activity in P. aeruginosa. (A) RhlR activity in the P. aeruginosa RhlR reporter strain PAO-SC4 (pPROBE-P_rhlA_) harboring plasmid-borne, arabinose-inducible *rhlR* (pJNR) (triangles and circles) or an empty vector control (pJN) (squares). Treatment with arabinose (ara) and/or 3OC12-HSL (10 µM) is indicated. Data are the means and ranges of two biological replicates and are representative of three independent experiments. (B) EC_50_ of 3OC12-HSL for LasR and QscR and of C4-HSL for RhlR when the indicated receptor is overexpressed (via pJNL, pJNQ, or pJNR) in the synthase-null P. aeruginosa mutant (PAO-SC4). EC_50_ was measured via the activity reporters pPROBE-P_rhlA_, -P_rsaL_, and -P_PA1897_, for RhlR, LasR, and QscR, respectively. Values are means and SEM from *n* ≥ 3 independent experiments. (C) Relative fluorescence (in relative fluorescence units [RFU]) of the synthase-null P. aeruginosa mutant (PAO-SC4) harboring the RhlR activity reporter pPROBE-P_rhlA_ and plasmid-borne, arabinose-inducible *rhlR* (pJNR) or an empty vector control (pJN). Treatment with 3OC12-HSL (10 µM) and/or arabinose is indicated. (D) Relative fluorescence of the synthase-null P. aeruginosa mutant (PAO-SC4) harboring the LasR activity reporter pPROBE-P_rsaL_ or the QscR activity reporter pPROBE-P_PA1897_ and plasmid-borne, arabinose-inducible *lasR* (pJNL) or *qscR* (pJNQ) or an empty vector (e) control (pJN). Treatment with arabinose is indicated. (E and F) Activation of QscR by a panel of 19 AHL signals (100 µM) measured via pPROBE-P_PA1897_ in the synthase-null P. aeruginosa mutant (PAO-SC4) harboring plasmid-borne, arabinose-inducible *qscR* (pJNQ) (F) or an empty vector control (pJN) (E). Activation is normalized to C10-HSL. Arabinose was added in both conditions. In panels C to F, bars are means and SEM from three independent experiments.

Multiple factors could account for the differences in sensitivity and selectivity between native and E. coli reporter methods. For example, efflux pumps have been demonstrated to decrease the sensitivity of LasR to 3OC12-HSL in P. aeruginosa (46). Additionally, many QS-controlled products are under complex regulation in their native organisms, with factors such as nutritional cues, phase of growth, negative regulators, and other QS receptors affecting their expression (33, 47–49). Given the previously reported effects of overexpression on AHL receptor sensitivity and selectivity (19, 25, 26), we hypothesized that high expression levels of the receptors may contribute to their increased promiscuity in E. coli reporters. To test this hypothesis, we introduced the plasmids carrying arabinose-inducible receptor genes used in the E. coli reporters into our P. aeruginosa reporter strains. Even without the addition of arabinose, the presence of the receptor expression plasmid resulted in increased P_rhlA_ activity and increased RhlR sensitivity to C4-HSL (Fig. 4A), likely due to leaky expression from the P_araBAD_ promoter. We observed a similar outcome for QscR, but not for LasR (Fig. S6A to C). For all three receptors, arabinose-induced overexpression from the P_araBAD_ promoter was sufficient to significantly increase the receptor’s sensitivity to its cognate AHL (Fig. 4A and B and Fig. S6A to C).

10.1128/mBio.00146-19.6 FIG S6 Effect of overexpression on AHL receptor activity in P. aeruginosa. (A) Activation of the P. aeruginosa LasR reporter (PAO-SC4 (pPROBE-P_rsaL_)) harboring plasmid-borne, arabinose-inducible *lasR* (pJNL) grown with arabinose (black circles) or without arabinose (open circles) or harboring an empty vector control (pJN) and grown with arabinose (gray squares). (B) Activation of the P. aeruginosa QscR reporter (PAO-SC4 (pPROBE-P_PA1897_)) harboring plasmid-borne, arabinose-inducible *qscR* (pJNQ) grown with arabinose (black circles) or without arabinose (open circles) or harboring an empty vector control (pJN) and grown with arabinose (gray squares). (C) As in panel B with axes altered to better show activation of the QscR reporter with the empty vector control (PAO-SC4 (pPROBE-P_PA1897_, pJN)). Data are the means and ranges of two (A) or three (B and C) biological replicates and are representative of three independent experiments. (D) Relative fluorescence (RFU) of the synthase-null P. aeruginosa *pqsE* deletion mutant (PAO-SC4Δ*pqsE*) harboring the RhlR activity reporter pPROBE-P_rhlA_ and plasmid-borne, arabinose-inducible *rhlR* (pJNR) or an empty vector control (pJN). Treatment with 3OC12-HSL (10 µM) and/or arabinose is indicated. Data are the means and SEM from four independent experiments. Download FIG S6, TIF file, 0.5 MB. Copyright © 2019 Wellington and Greenberg. 2019 Wellington and Greenberg This content is distributed under the terms of the Creative Commons Attribution 4.0 International license.

Surprisingly, receptor overexpression in P. aeruginosa resulted in AHL-independent activity for all three receptors (Fig. 4C and D). This effect was particularly large for QscR and RhlR. Addition of 3OC12-HSL also resulted in C4-HSL-independent RhlR activity (Fig. 4C). Because 3OC12-HSL does not affect RhlR activity in E. coli (Fig. S3), this observed activity is likely due to upregulation of *rhlR* by LasR rather than to 3OC12-HSL binding to RhlR. PqsE, a thioesterase that is part of the *Pseudomonas* quinolone signal (PQS) operon (50), is also known to positively affect RhlR activity (51–53) and was recently suggested to be the synthase of an alternative RhlR ligand (54). To determine whether our observed AHL-independent RhlR activity requires PqsE, we deleted *pqsE* in AHL synthase-null P. aeruginosa (PAO-SC4) and measured RhlR activity via the P_rhlA_-*gfp* reporter. *rhlR* overexpression in strain PAO-SC4Δ*pqsE* resulted in C4-HSL-independent activation of the *rhlA* promoter comparable to that observed in strain PAO-SC4 (Fig. S6D).

To determine the effect of receptor overexpression on apparent promiscuity, we measured QscR activation by our panel of AHLs. Overexpression of *qscR* in our P. aeruginosa reporter strain PAO-SC4 (pPROBE-P_PA1897_) dramatically increased the receptor’s response to numerous signals (Fig. 4E and F) such that it appears even more promiscuous than in the E. coli QscR reporter (Fig. S3). Overexpression is therefore sufficient to significantly increase sensitivity and promiscuity.

## DISCUSSION

AHL QS has long been recognized as a form of intraspecies bacterial communication. There are, however, examples of “promiscuous” AHL signal receptors, which are able to respond to signals other than their self-produced cognate AHL (8, 9). Existing data on QS selectivity are limited and often generated by E. coli reporter methods in which overexpression of the receptor may artificially enhance promiscuity. To better understand the prevalence and potential function and evolution of promiscuous QS receptors, we systematically studied the selectivity of AHL receptors in their native host organisms. To compare our results with previous studies, we also constructed reporters of receptor activity using heterologous expression in E. coli. The E. coli reporters consistently overestimated receptor sensitivity and promiscuity. Further, we found that overexpression of the AHL receptors in P. aeruginosa was sufficient to increase receptor sensitivity and promiscuity to levels equal to or greater than those of the E. coli reporters. Transcription of the target DNA in our reporter assays is a reflection of many processes, including AHL receptor stability, receptor dimerization, and, ultimately, receptor binding to DNA. AHL binding both stabilizes receptors by promoting proper folding and protecting them against proteolysis and promotes receptor dimerization and binding to DNA (55–57). When considering activity in our reporter assays as a reflection of a binding reaction, receptor + AHL ⇌ receptor · AHL, the concentration of the receptor-AHL complex, and therefore the activity of the reporter, is dependent on both the concentration of AHL ([AHL]) and [receptor] in addition to the affinity of the receptor for the AHL. This fundamental principle of protein-ligand interactions can explain how increased expression of the receptor increases the sensitivity of the activity reporter to both cognate and noncognate AHLs. Indeed, changes in receptor expression and/or stability have been linked to generalized changes in receptor sensitivity previously (19, 25, 26). Importantly, AHL receptor expression is typically affected by complex regulatory systems, and receptor expression levels can vary between strains and between environmental conditions (58, 59). It is likely that this variability in expression level leads to variable AHL receptor responses to both self and nonself signals in natural systems.

Surprisingly, we also found that receptor overexpression in P. aeruginosa results in AHL-independent activity. Although it is possible that a non-AHL small molecule is responsible for the observed activity (54, 60), it is also possible that artificially high expression of the receptors could drive ligand-free DNA binding in our system. Because PqsE was recently suggested to be the synthase of an alternative RhlR ligand (54), we tested the effect of *pqsE* deletion on RhlR activity. In our *pqsE* deletion strain, *rhlR* overexpression still resulted in AHL-independent RhlR activity. Given this finding and given that all three receptors (RhlR, LasR, and QscR) displayed AHL-independent activity when overexpressed in P. aeruginosa, we favor ligand-free activation as an explanation for our observed AHL-independent activity. AHL receptors are typically unstable in the absence of an AHL and require AHL for binding to promoters *in vitro* (20, 55, 57, 61). However, some receptors, such as RhlR (54), are more stable in their parent strains than when expressed in E. coli or purified, and further, some orphan/solo AHL receptor homologs are able to exert AHL-independent control over their regulons (38, 62). Perhaps increased expression of the P. aeruginosa receptors produces sufficient quantities of stable receptor to promote some degree of ligand-free DNA binding in the parent strain.

E. coli reporter methods, of course, have important applications. First, the QS systems of many bacteria have not been studied well enough to construct reporters of receptor activity in the natural host organism. E. coli methods can also remove confounding factors that arise from the complex natural regulation of QS systems and their products. Some caution must be applied, however. The molecular mechanisms that modulate the activity of QS receptors and their regulons in natural host organisms are sometimes present and functional in E. coli as well (63). Additionally, E. coli has an AHL receptor, SdiA, which can interfere with receptor activity studies by activating transcription from the target promoter (64, 65). Although some researchers use *sdiA* deletion strains (43, 66), it is common to use readily available chemically competent cells such as TOP10 or, as we have, DH5α which have intact *sdiA* and the potential for artifacts associated with this receptor. We note, however, that in our E. coli experiments, activity of the reporter required expression of the receptor of interest and was, therefore, unlikely to be affected by SdiA. Finally, our findings highlight that results from any study using artificial expression of QS receptors should be interpreted with their limitations in mind, namely, the artifacts of increased receptor sensitivity and promiscuity, and the potential for ligand-independent activity. These findings may also inform the design of engineered cell circuits where it is important to limit cross talk between receptors and where AHL-independent activation of receptors may have detrimental effects on applications in engineered biosensors and targeted therapeutic delivery systems (67).

By systematically and quantitatively measuring receptor responses in their natural backgrounds, we found that AHL QS receptors display a wide range of signal preferences and selectivity. Some AHL receptors, such as RhlR, are highly specific for their cognate signal. Because it is highly conserved across P. aeruginosa clinical isolates and is essential for virulence in animal models, RhlR has emerged as a potential antivirulence therapeutic target for P. aeruginosa (60, 66, 68, 69). Encouragingly, RhlR’s specific detection of C4-HSL may be advantageous for the development of selective RhlR inhibitors. Our finding that RhlR activity is not saturated even at 1 mM C4-HSL is somewhat surprising, but it is consistent with previous studies where high concentrations of C4-HSL were required for maximal activity (23, 66, 69). The relative shallowness of RhlR’s dose-response curve to C4-HSL could allow for a greater ability to modulate activity of the RhlR regulon in nature. In laboratory cultures, clinical isolates of P. aeruginosa can make as much as fivefold more C4-HSL than the laboratory strain PAO1 and can also produce larger amounts of various RhlR-regulated products (68), possibly due to altered gene regulation and/or increased C4-HSL production.

On the other end of the spectrum, certain receptors, such as BtaR2, are very promiscuous, responding to nanomolar concentrations of several signals. The selectivity of the rest of the AHL receptors lies on a continuum, with most receptors responding strongly and sensitively to at least one noncognate signal. Using these data, we can begin to make testable hypotheses about cross talk between QS systems in natural polymicrobial communities. In the context of a QS proficient strain, promiscuous activation by a noncognate signal often results in early activation of the QS receptor (i.e., activation at lower cell densities) (8, 9, 11). It is also important to consider that noncognate AHLs can inhibit receptor activation by cognate AHLs through various mechanisms, including partial agonism, receptor destabilization, and stabilization of the receptor in an inactive conformation (7, 15, 16, 18, 19, 22, 44). As with activation, the receptors in our study were variably sensitive to inhibition. We and others have found that LuxR and CviR are sensitive to inhibition by noncognate AHLs (7, 15, 16, 18). In our study, LasR and QscR were less sensitive to inhibition. Previous studies have reported more significant inhibition of LasR activity by noncognate AHLs, with some AHLs acting as inhibitors at lower concentrations and as agnoists at higher concentrations (26, 46). Therefore, we may have missed the inhibitory activity of some AHLs by measuring at a single concentration. In any case, it is clear that AHL receptors are susceptible to inhibition by noncognate AHLs and that there are likely a multitude of complex positive and negative interactions mediated by AHLs in natural polymicrobial communities.

Our quantitative scoring of receptor selectivity also serves as a basis for hypotheses about the benefits of specific versus promiscuous QS activation and about how the diversity in QS signals and receptors evolved. With the exception of QscR, which has no cognate signal, all receptors tested were most sensitive (i.e., they respond with the lowest EC_50_) to their self-produced cognate signal. By amino acid sequence, QscR is more closely related to AHL receptors from other organisms than to the other P. aeruginosa receptors, LasR and RhlR (70). QscR has, therefore, been hypothesized to have been introduced to P. aeruginosa via horizontal gene transfer (20). It is possible that QscR originates from a C10-HSL-responsive receptor and has evolved additional 3OC12-HSL recognition. Alternatively, promiscuous QscR activation could have arisen due to some selective advantage for P. aeruginosa.

Interestingly, the two most promiscuous receptors, CviR and BtaR2, control the synthesis of potent antibiotics, violacein (7) and bactobolin (21), respectively. We have previously shown that promiscuous activation of CviR can confer a competitive advantage to C. violaceum due to QS-controlled antimicrobial production (8). We speculate that the selectivity of each AHL receptor may be dictated by its regulon, i.e., by a selective advantage gained from either specific or promiscuous receptor activation, and/or by the evolutionary history of the receptor. We are just beginning to understand the large diversity in AHL receptor structure and function. To gain insight into the origin and function of promiscuous signaling, it will be necessary to determine what, if any, impact exists for the promiscuous activation of each receptor. Our comprehensive data set on AHL receptor selectivity gives us a rational basis to begin to address longstanding questions about how the diverse array of AHL signal synthase-receptor pairs has evolved in proteobacteria and about how signaling systems interact in nature.

## MATERIALS AND METHODS

### Bacterial strains, plasmids, and culture conditions.

Bacteria and plasmids are listed in Tables S1 and S2 in the supplemental material. V. fischeri was grown in Sea Water Complete (SWC) broth (5 g tryptone, 3 g yeast extract per liter in 75% seawater, 25% tap water, 0.3% glycerol [pH 7.0]) or on SWC agar (SWC broth plus 1.5% Bacto agar). All other bacteria were grown in lysogeny broth (LB) (10 g tryptone, 5 g yeast extract, 5 g NaCl per liter) with 50 mM 3-(*N*-morpholino)propanesulfonic acid (MOPS) (pH 7) or on LB agar (LB plus 1.5% Bacto agar) supplemented as noted. Antibiotics used for selection and plasmid maintenance were as follows: for E. coli, 100 µg/ml ampicillin (Ap) and 10 µg/ml gentamicin (Gm); for P. aeruginosa, 200 µg/ml carbenicillin (Cb) and 30 µg/ml Gm; for B. thailandensis, 450 µg/ml Gm. Where indicated, L-arabinose was added at a final concentration of 0.4% (wt/vol). AHLs were obtained from commercial sources (Sigma-Aldrich, Cayman Chemical Company, and the University of Nottingham quorum sensing research group) and used without further purification. AHLs were dissolved in ethyl acetate acidified with 0.01% glacial acetic acid, and prior to addition of bacterial cells, the solution was dried on the bottom of the culture vessel. Bacteria were grown at room temperature (V. fischeri), 30°C (C. violaceum), or 37°C (E. coli, P. aeruginosa, and B. thailandensis) with shaking.

### Strain and plasmid construction.

pPROBE-GT vectors were constructed as follows. PCR fragments were generated with SalI (P_rsaL_, P_PA1897_, and P_cdiA_) or HindIII (P_btaK_) and BamHI restriction sites flanking the promoter region. PCR fragments and pPROBE-GT were digested with restriction enzymes and then were ligated using T4 DNA ligase. Assembled plasmids were used to transform E. coli and verified by Sanger sequencing.

To construct arabinose-inducible expression plasmids of *lasR*, *rhlR*, *qscR*, and *btaR2*, we swapped the Gm resistance cassette from pJN105L, pJN105.*rhlR*, pJN105Q, and pJNR2 to Ap resistance by E. coli-mediated assembly of DNA fragments with end homologies (71). Briefly, the *bla* gene and the pJN105 vector were PCR amplified with primers designed to add 17 to 24 bases of homology between the two sequences. NEB 5-alpha E. coli cells were then cotransformed with purified PCR products. The BtaR1 expression plasmid, pJNR1, was constructed as follows. *btaR1* was amplified from B. thailandensis E264 gDNA using primers that added EcoRI and XbaI restriction sites. Both the PCR product and pJN were digested and then ligated to each other.

To create a chromosomal integration of the P_rsaL_-*gfp* reporter construct, we PCR amplified pUC18mini-Tn7T-Gm by using the primers 5′CGGCCCCGTACCCAGCTTTTGCCTCGCGAAGGCCTTGCAGGCC and 5′GCCTGGAATTGGGAATTGCGGCTTCTCGAGGAATTCCTGCAG and amplified pPROBE-P_rsaL_ with the reverse complement of the same primers such that the resulting PCR fragment contained the four terminators upstream of P_rsaL_, the promoter-*gfp* sequence, and the terminator downstream from *gfp*. The PCR primers introduced regions of homology such that the reporter fragment would ligate to the linear pUC18mini-Tn7T-Gm PCR product at the multiple cloning site (MCS) using E. coli-mediated assembly of DNA fragments with end homologies (71). We cotransformed into P. aeruginosa PAO-SC4 pUC18mini-Tn7T-P_rsaL_-*gfp* and pTNS3 to facilitate insertion of the reporter construct into the neutral *att*Tn*7* site (72). Insertions were verified using the primer pairs P*_glmS_*_-down_-P_Tn7R_ and P*_glmS_*_-up_-P_Tn7L_ (72).

Plasmids were introduced into E. coli by using heat shock and into P. aeruginosa and B. thailandensis by electroporation.

*pqsE* was deleted from *P*. *aeruginosa* PAO-SC4 using methods that will be reported (M. Kostylev, D. Kim, N. E. Smalley, I. Salukhe, E. P. Greenberg, and A. A. Dandekar, submitted for publication).

### Receptor activity measurements.

All experiments were begun with stationary-phase overnight-grown starter cultures. The starter cultures were diluted 1:100 and grown back to log phase (optical density at 600 nm [OD_600_] between 0.05 and 0.3). All native reporters were diluted to an OD_600_ of 0.01 and then incubated in 96-well deep well plates with AHLs at the indicated concentrations for 16 to 18 h (C. violaceum, P. aeruginosa, and B. thailandensis) or for 6 h (V. fischeri). E. coli reporters were treated as previously reported (73), with the exception that incubation time was extended to 4 h. Briefly, E. coli reporters were grown to an OD_600_ of 0.3, then arabinose was added to induce receptor expression, and cultures were incubated with AHLs for 4 h. Incubation times were selected to ensure robust reporter activity for all receptors.

For all bacteria except C. violaceum, following incubation, 100 µl of each culture was transferred to a black 96-well plate with a clear bottom to measure OD_600_ and activation using a Synergy H1 microplate reader (BioTek Instruments). Activation of LuxR in V. fischeri (MJ215) was quantified by measuring luminescence. Activation of receptors in P. aeruginosa, B. thailandensis, and E. coli was measured as GFP fluorescence (excitation 490 nm, emission 520 nm, gain 50). Activation measurements were normalized by dividing by OD_600_.

Violacein was used as a measure of CviR activity in C. violaceum (CV026). Briefly, overnight cultures were pelleted by centrifugation and suspended in an equal volume of DMSO to extract the violacein. Cells were pelleted and 100 µl supernatant fluid was transferred to a clear 96-well plate. Violacein was quantified by measuring absorbance at 585 nm (74).

### Selectivity score.

The area under the curve (AUC) for the activation of each receptor by each signal was calculated using GraphPad Prism for AHL concentrations up to 100 µM. Selectivity was calculated using the following formula:

$$\text{selectivity score}=\frac{{\text{AUC}}_{\text{Most}\;\text{potent}\;\text{AHL}}}{\Sigma {\;\text{AUC}}_{\text{All}\;\text{other}\;\text{AHLs}}}$$

Reported scores are means ± standard deviations of *n* ≥ 3 independent experiments.
