# Supplementary material for: Quorum Sensing Signal Selectivity and the Potential for Interspecies Cross Talk
Source: mBio. 2019 Mar 5;10(2):e00146-19. doi: 10.1128/mBio.00146-19 (PMC6401477; doi:10.1128/mBio.00146-19)
Supplement: TABLE S1 [file mBio.00146-19-st001.docx]

**Table S1.** Bacterial strains used in this study

| Strain | Description | Source |
| --- | --- | --- |
| *Vibrio fischeri* MJ215 | AHL synthase-null mutant (∆*luxI*∆*ainS*) | (1) |
| *Chromobacterium violaceum* CV026 | Double transposon mutant, AHL negative | (2) |
| *Pseudomonas aeruginosa* PAO1 | Wild-type strain | (3) |
| PAO-SC4 | AHL synthase-null mutant; PAO1 with unmarked deletions of *lasI* and *rhI* | ^a^ |
| PAO-SC4 (pPROBE-P_rsaL_) | LasR reporter; PAO-SC4 w/ pPROBE-P_rsaL_ | This study |
| PAO-SC4-P_rsaL_-*gfp* | Chromosomal LasR reporter; PAO-SC4 with P_rsaL_-*gfp* in the chromosome | This study |
| PAO-SC4 (pPROBE-P_rhlA_) | RhlR reporter; PAO-SC4 w/ pPROBE-P_rhlA_ | This study |
| PAO-SC4 (pPROBE-P_PA1897_) | QscR Reporter; PAO-SC4 with pPROBE-P_PA1897_ | This study |
| PAO-SC4 (pPROBE-P_rsaL_, pJN) | PAO-SC4 with pPROBE-P_rsaL_ and pJN | This study |
| PAO-SC4 (pPROBE-P_rsaL,_ pJNL) | PAO-SC4 with pPROBE-P_rsaL_ and pJNL | This study |
| PAO-SC4 (pPROBE-P_rhlA_, pJN) | PAO-SC4 with pPROBE-P_rhlA_ and pJN | This study |
| PAO-SC4 (pPROBE-P_rhlA_, pJNR) | PAO-SC4 with pPROBE-P_rhlA_ and pJNR | This study |
| PAO-SC4 (pPROBE-P_PA1897_, pJN) | PAO-SC4 with pPROBE-P_PA1897_ and pJN | This study |
| PAO-SC4 (pPROBE-P_PA1897_, pJNQ) | PAO-SC4 with pPROBE-P_PA1897_ and pJNQ | This study |
| PAO-SC4∆*pqsE* | PAO-SC4 with an unmarked deletion of *pqsE* | This study |
| PAO-SC4∆*pqsE* (pPROBE-P_rhlA_, pJN) | PAO-SC4∆*pqsE* with pPROBE-P_rhlA_ and pJN | This study |
| PAO-SC4∆*pqsE* (pPROBE-P_rhlA_, pJNR) | PAO-SC4∆*pqsE* with pPROBE-P_rhlA_ and pJNR | This study |
| *Burkholderia thailandensis* E264 | Wild-type strain |  |
| JBT112 | AHL synthase-null mutant; E264∆*btaI1*∆*btaI2*∆*btaI3* | (4) |
| JBT107 | E264∆*btaR1* | (4) |
| JBT108 | E264∆*btaR2* | (4) |
| JBT112 (pPROBE-P_cdiA_) | BtaR1 reporter; JBT112 w/ pPROBE-P_cdiA_ | This study |
| JBT112 (pPROBE-P_btaK_) | BtaR2 reporter; JBT112 w/ pPROBE-P_btaK_ | This study |
| JBT107 (pPROBE-P_cdiA_) | JBT107 with pPROBE-P_cdiA_ | This study |
| JBT108 (pPROBE-P_btaK_) | JBT108 with pPROBE-P_btaK_ | This study |
| E264 (pPROBE-P_cdiA_) | E264 with pPROBE-P_cdiA_ | This study |
| E264 (pPROBE-P_btaK_) | E264 with pPROBE-P_btaK_ | This study |
| *Escherichia coli* | NEB 5α |  |
| 5α (pJNL, pPROBE-P_rsaL_) | *E. coli* LasR reporter; 5α harboring pJNL and pPROBE-P_rsaL_ | This study |
| 5α (pJNR, pPROBE-P_rhlA_) | *E. coli* RhlR reporter; 5α harboring pJNR and pPROBE-P_rhlA_ | This study |
| 5α (pJNQ, pPROBE-P_PA1897_) | *E. coli* QscR repoter; 5α harboring pJNQ and pPROBE-P_PA1897_ | This study |
| 5α (pJNR1, pPROBE-P_cidA_) | *E. coli* BtaR1 reporter; 5α harboring pJNR1 and pPROBE-P_cdiA_ | This study |
| 5α (pJNR2, pPROBE-P_btaK_) | *E. coli* BtaR2 reporter; 5α harboring pJNR2 and pPROBE-P_btaK_ | This study |

^a^This strain was a gift from S. Chugani and E.P. Greenberg, generated using previously published methods (5).

**REFERENCES**

1. Kuo A, Callahan SM, Dunlap PV. 1996. Modulation of luminescence operon expression by N-octanoyl-L-homoserine lactone in *ainS* mutants of *Vibrio fischeri*. J Bacteriol 178: 971-976.

2. McClean KH, Winson MK, Fish L, Taylor A, Chhabra SR, Camara M, Daykin M, Lamb JH, Swift S, Bycroft BW, Stewart GSAB, Williams P. 1997. Quorum sensing and *Chromobacterium violaceum*: Exploitation of violacein production and inhibition for the detection of N-acylhomoserine lactones. Microbiology 143: 3703-3711.

3. Stover CK, Pham XQ, Erwin AL, Mizoguchi SD, Warrener P, Hickey MJ, Brinkman FSL, Hufnagle WO, Kowalik DJ, Lagrou M, Garber RL, Goltry L, Tolentino E, Westbrock-Wadman S, Yuan Y, Brody LL, Coulter SN, Folger KR, Kas A, Larbig K, Lim R, Smith K, Spencer D, Wong GKS, Wu Z, Paulsen IT, Reizer J, Saier MH, Hancock REW, Lory S, Olson MV. 2000. Complete genome sequence of *Pseudomonas aeruginosa* PAO1, an opportunistic pathogen. Nature 406: 959-964.

4. Chandler JR, Duerkop BA, Hinz A, West TE, Herman JP, Churchill MEA, Skerrett SJ, Greenberg EP. 2009. Mutational analysis of *Burkholderia thailandensis* quorum sensing and self-aggregation. J Bacteriol 191: 5901-5909.

5. Chugani S, Greenberg EP. 2010. LuxR homolog-independent gene regulation by acyl-homoserine lactones in *Pseudomonas aeruginosa*. Proc Natl Acad Sci U S A 107: 10673-10678.
