# Supplementary figures and images for: Quorum Sensing Signal Selectivity and the Potential for Interspecies Cross Talk
Source: mBio. 2019 Mar 5;10(2):e00146-19. doi: 10.1128/mBio.00146-19 (PMC6401477; doi:10.1128/mBio.00146-19)

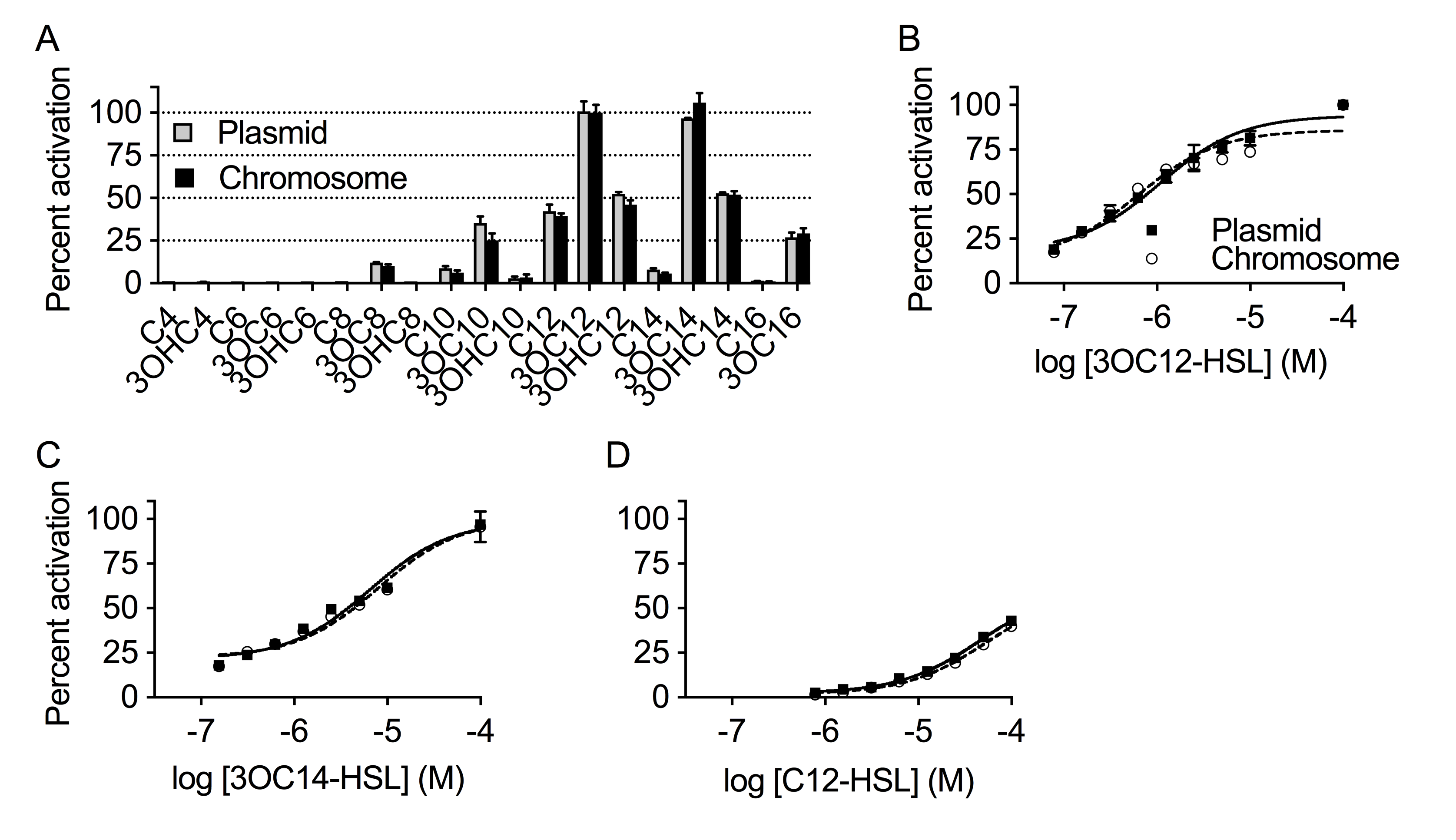

Supplement: FIG S1 [file mBio.00146-19-sf001.tif]

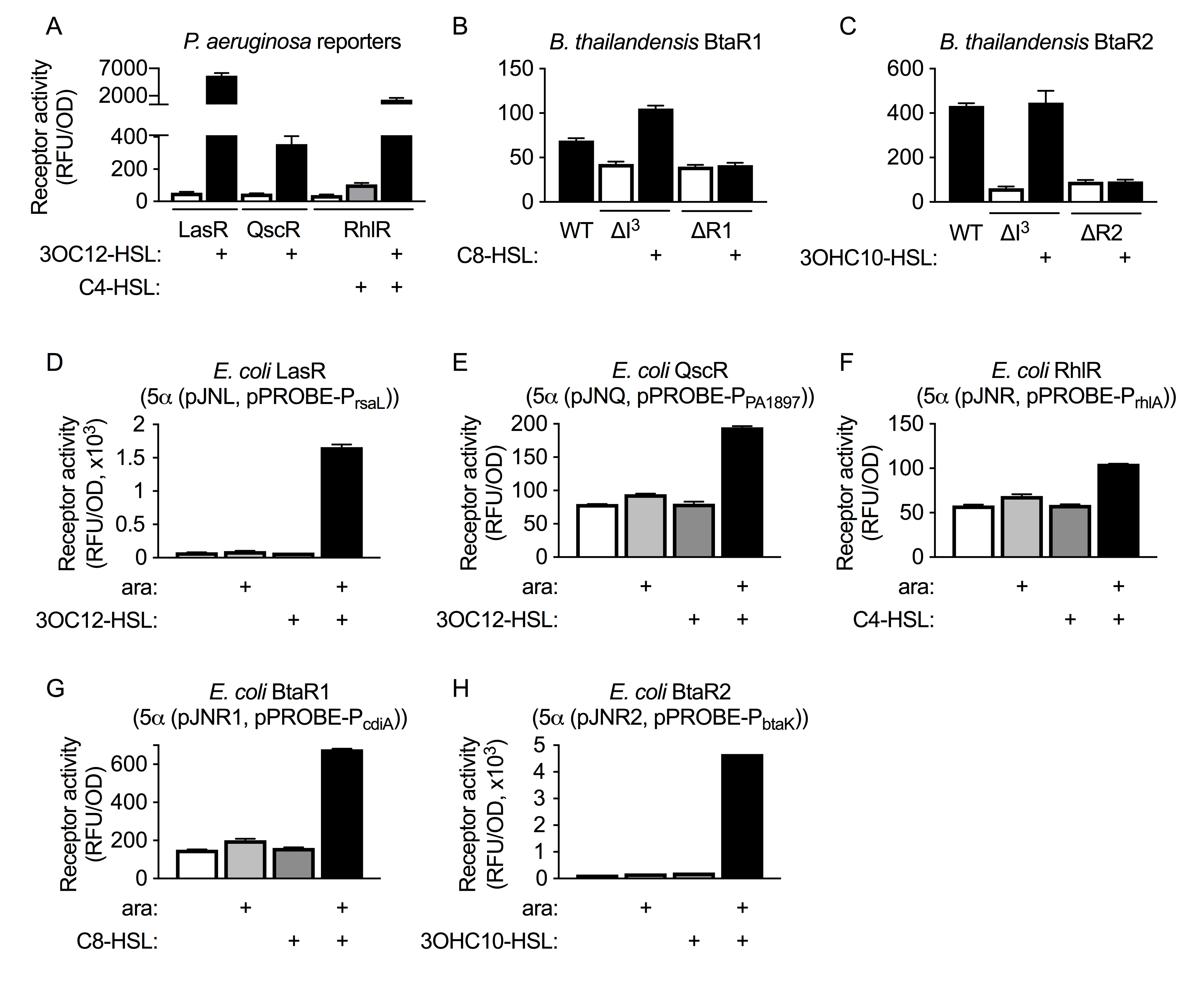

Supplement: FIG S2 [file mBio.00146-19-sf002.tif]

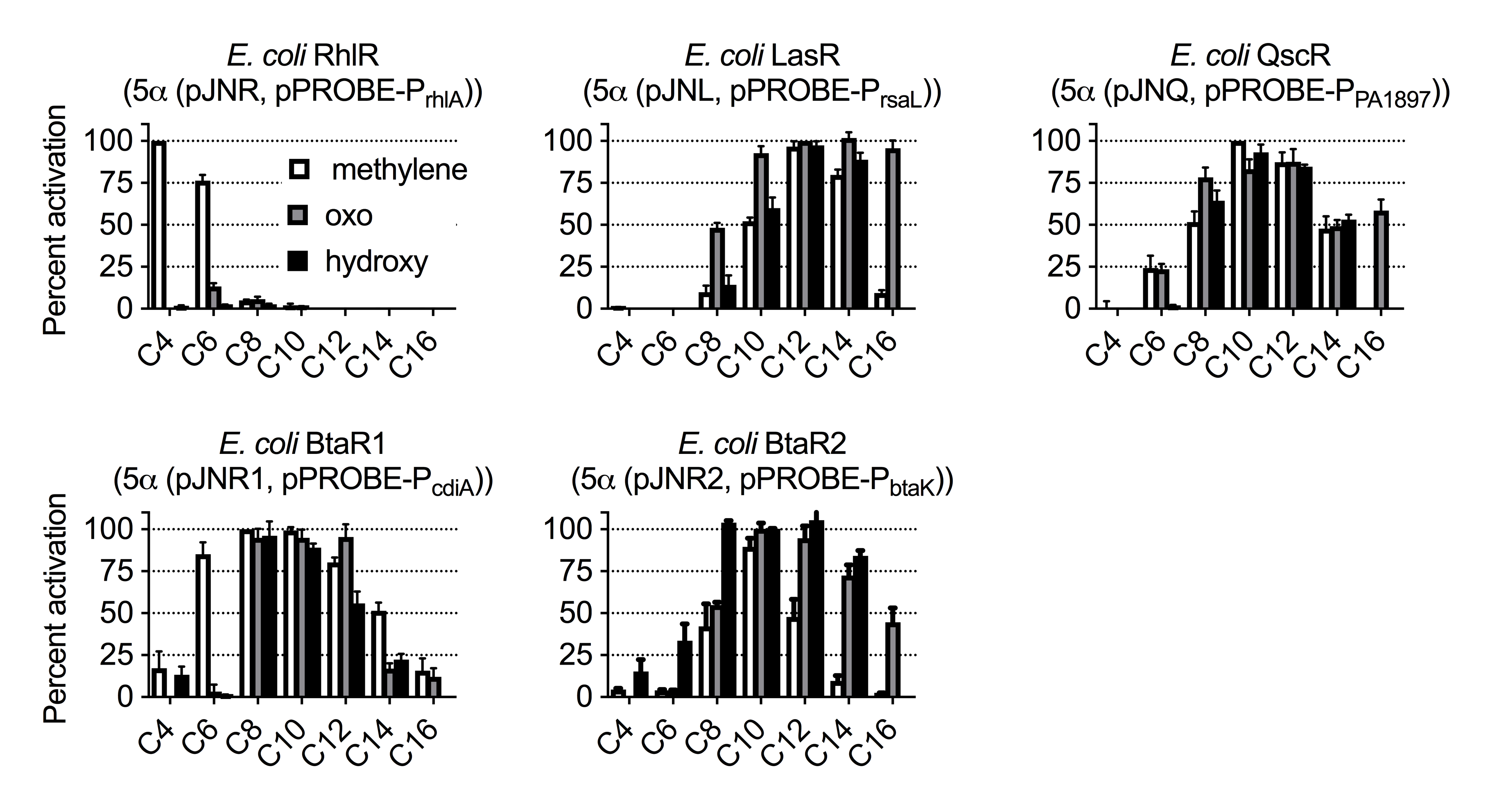

Supplement: FIG S3 [file mBio.00146-19-sf003.tif]

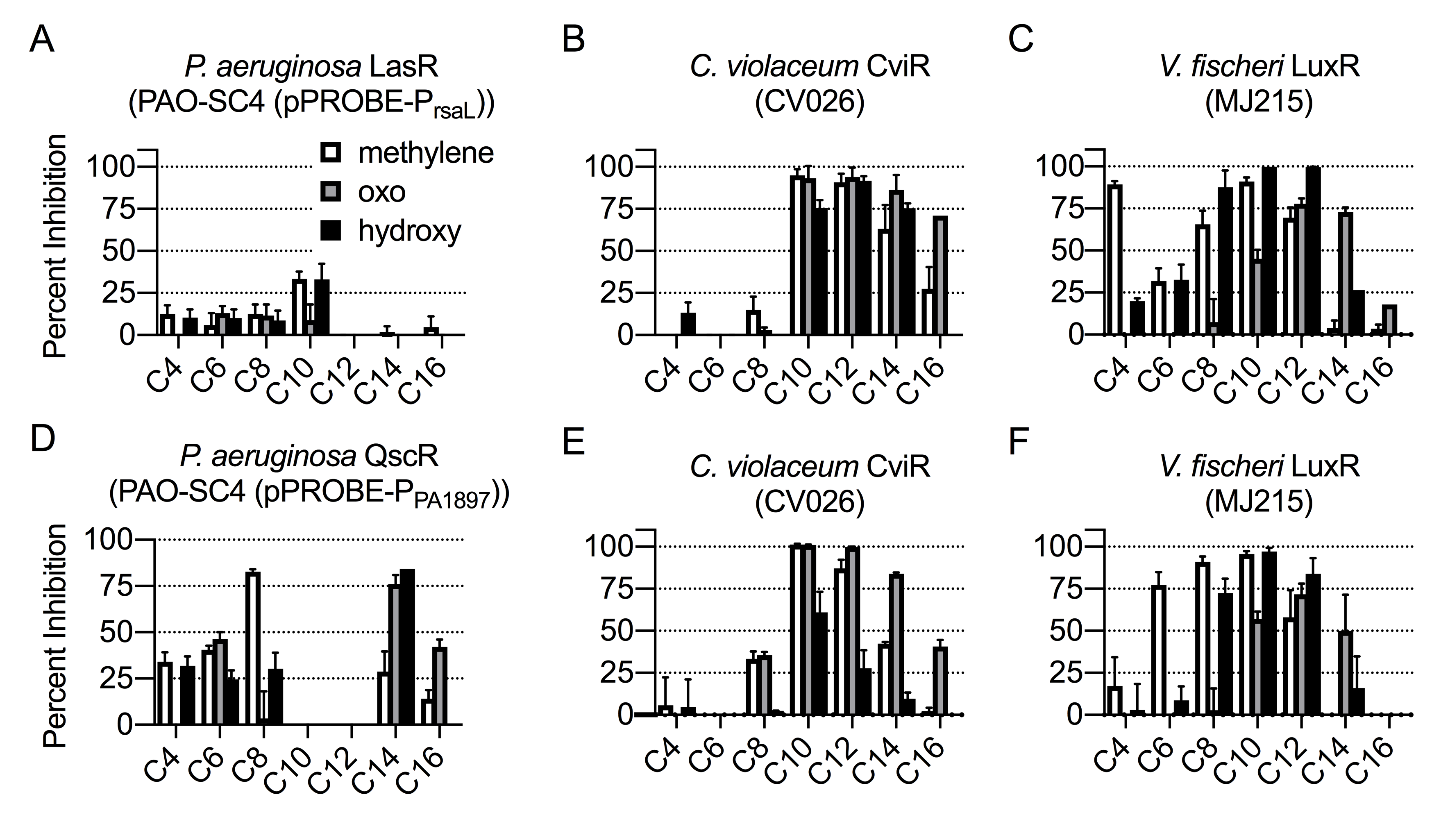

Supplement: FIG S4 [file mBio.00146-19-sf004.tif]

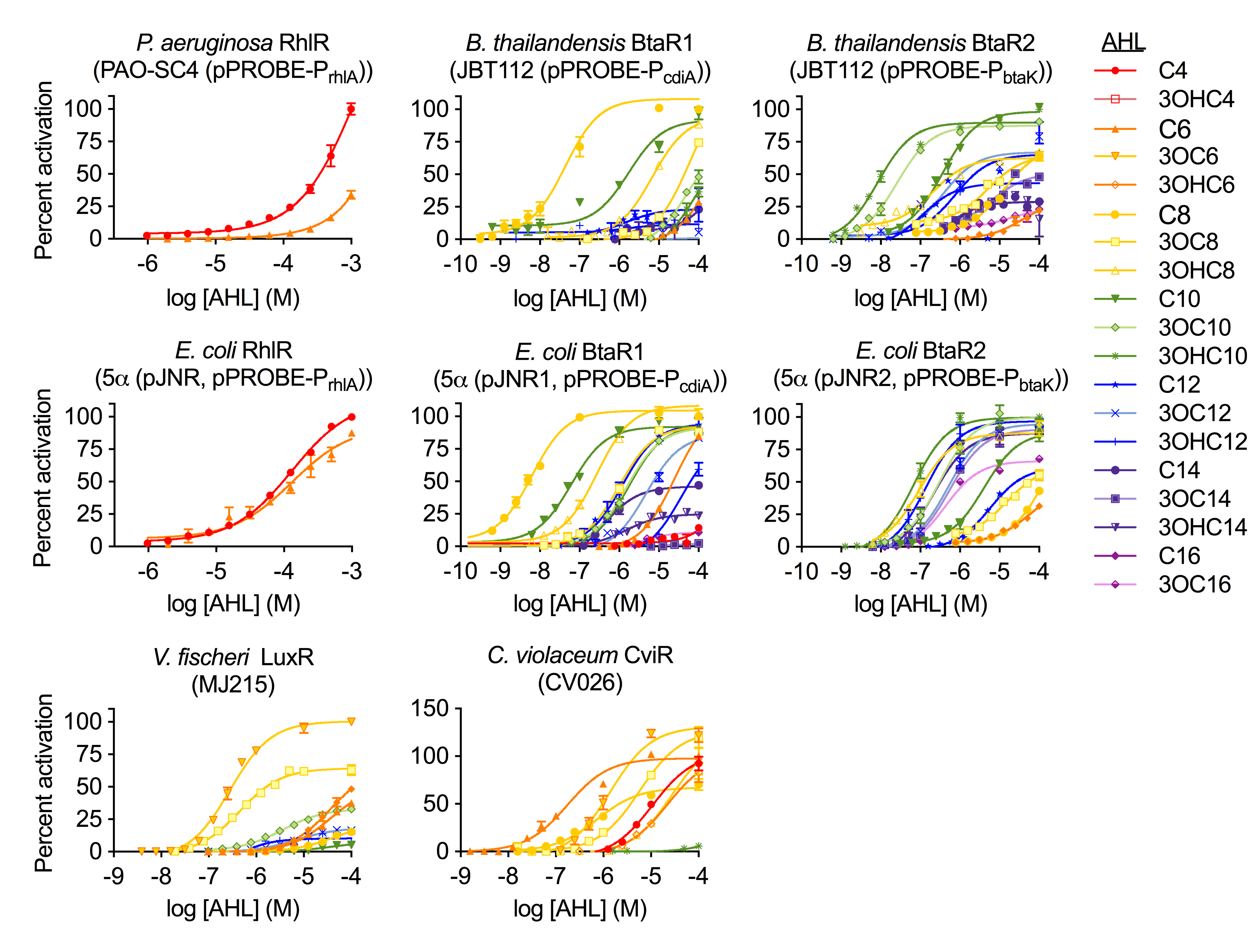

Supplement: FIG S5 [file mBio.00146-19-sf005.tif]

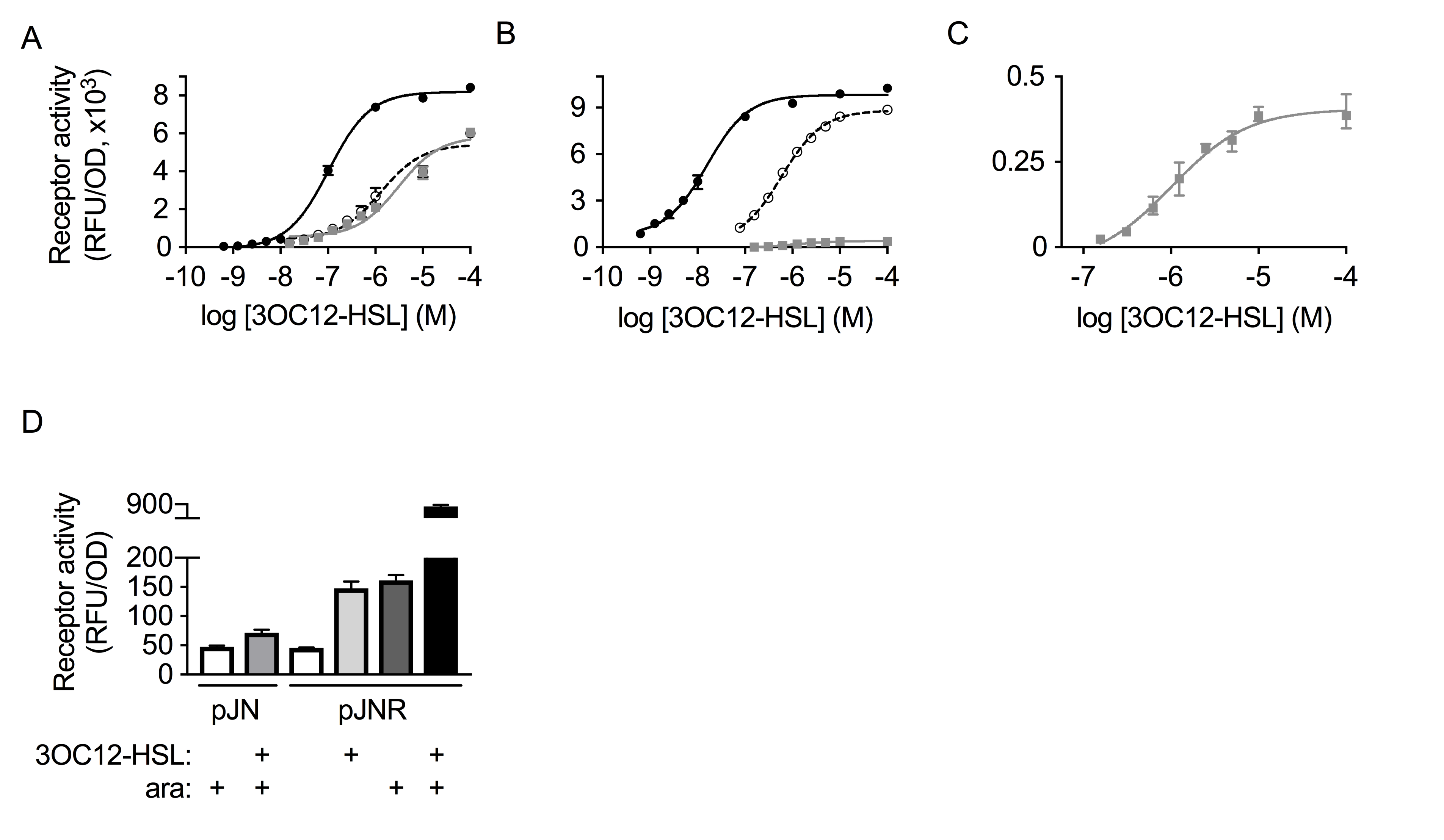

Supplement: FIG S6 [file mBio.00146-19-sf006.tif]
