# Supplementary material for: Quorum Sensing Signal Selectivity and the Potential for Interspecies Cross Talk
Source: mBio. 2019 Mar 5;10(2):e00146-19. doi: 10.1128/mBio.00146-19 (PMC6401477; doi:10.1128/mBio.00146-19)
Supplement: TABLE S2 [file mBio.00146-19-st002.docx]

**Table S2.** Plasmids used in this study

| Plasmid | Description | Source |
| --- | --- | --- |
| pPROBE-P_rsaL_ | pPROBE-GT (1) with the *rsaL* promoter extending from  -290 to +103, Gm^r^ | ^a^ |
| pPROBE-P_rhlA_ | pPROBE-GT with the *rhlA* promoter, Gm^r^ | (2) |
| pPROBE-P_PA1897_ | pPROBE-GT with the *pa1897* promoter extending from  -300 to + 39 (3), Gm^r^ | This study |
| pPROBE-P_cdiA_ | pPROBE-GT with the *cdiA* (BTH_I2723) promoter extending from -500 to +100, Gm^r^ | This study |
| pPROBE-P_btaK_ | pPROBE-GT with the *btaK* (BTH_II1233) promoter extending form -409 to +13 (4), Gm^r^ | This study |
| pJN | Arabinose-inducible gene expression vector derived from pJN105 (5), Ap^r^ | This study |
| pJNL | *lasR* expression vector derived from pJN105L (3), Ap^r^ | This study |
| pJNR | *rhlR* expression vector derived from pJN105.*rhlR* (6), Ap^r^ | This study |
| pJNQ | *qscR* expression vector derived from pJN105Q (3), Ap^r^ | This study |
| pJNR1 | pJN with the *btaR1* (BTH_II1510) coding sequence extending from -18 to +720, Ap^r^ | This study |
| pJNR2 | *btaR2* expression vector derived from pJNR2 (4), Ap^r^ | This study |
| pUC18-mini-Tn7T-Gm | Suicide delivery vector, Gm^r^ | (7) |
| pUC18-miniTn7T-P_rsaL_-*gfp* | Suicide delivery vector containing P_rsaL_-*gfp* with upstream and downstream terminators from pPROBE-P_rsaL_, Gm^r^ | This study |
| pTNS3 | Helper plasmid with *tnsABCD* genes for Tn7 transposition pathway, Ap^r^ | (8) |

^a^This plasmid was a gift from R.L. Scholz and E.P. Greenberg, generated using previously published methods (9).

**REFERENCES**

1. Miller WG, Leveau JHJ, Lindow SE. 2000. Improved *gfp* and *inaZ* broad-host-range promoter-probe vectors. Mol Plant-Microbe Interact 13: 1243-1250.

2. Feltner JB, Wolter DJ, Pope CE, Groleau M-C, Smalley NE, Greenberg EP, Mayer-Hamblett N, Burns J, Déziel E, Hoffman LR, Dandekar AA. 2016. LasR variant cystic fibrosis isolates reveal an adaptable quorum-sensing hierarchy in *Pseudomonas aeruginosa*. mBio 7: e01513-01516.

3. Lee J-H, Lequette Y, Greenberg EP. 2006. Activity of purified QscR, a *Pseudomonas aeruginosa* orphan quorum-sensing transcription factor. Mol Microbiol 59: 602-609.

4. Duerkop BA, Varga J, Chandler JR, Peterson SB, Herman JP, Churchill MEA, Parsek MR, Nierman WC, Greenberg EP. 2009. Quorum-sensing control of antibiotic synthesis in *Burkholderia thailandensis*. J Bacteriol 191: 3909-3918.

5. Newman JR, Fuqua C. 1999. Broad-host-range expression vectors that carry the L-arabinose-inducible *Escherichia coli araBAD* promoter and the *araC* regulator. Gene 227: 197-203.

6. Schuster M, Greenberg EP. 2007. Early activation of quorum sensing in *Pseudomonas aeruginosa* reveals the architecture of a complex regulon. BMC Genomics 8: 287-287.

7. Choi K-H, Schweizer HP. 2006. mini-Tn7 insertion in bacteria with single *att*Tn7 sites: Example *Pseudomonas aeruginosa*. Nat Protoc 1: 153-161.

8. Choi K-H, Mima T, Casart Y, Rholl D, Kumar A, Beacham IR, Schweizer HP. 2008. Genetic tools for select-agent-compliant manipulation of *Burkholderia pseudomallei*. Appl Environ Microbiol 74: 1064-1075.

9. Scholz RL, Greenberg EP. 2017. Positive autoregulation of an acyl-homoserine lactone quorum-sensing circuit synchronizes the population response. mBio 8: e01079-e01117.
