# Supplementary material for: Quorum Sensing Signal Selectivity and the Potential for Interspecies Cross Talk
Source: mBio. 2019 Mar 5;10(2):e00146-19. doi: 10.1128/mBio.00146-19 (PMC6401477; doi:10.1128/mBio.00146-19)
Supplement: TABLE S3 [file mBio.00146-19-st003.docx]

**Table S3.** EC_50_ values (µM unless indicated) for receptors in their native hosts^a^

| AHL | LasR | RhlR | QscR | BtaR1 | BtaR2 | LuxR | CviR |
| --- | --- | --- | --- | --- | --- | --- | --- |
| C4 | — | >100 | — | — | — | — | 14.3 ± 3.8 |
| 3OHC4 | — | >100 | — | — | NR | — | — |
| C6 | — | NR | — | NR | NR | NR | 83.4 ± 24.5 nM |
| 3OC6 | — | NR | — | — | NR | 0.27 ± 0.01 | 0.98 ± 0.26 |
| 3OHC6 | — | — | — | — | NR | NR | NR |
| C8 | — | — | — | 50.5 ± 4.6 nM | 13.9 ± 2.1 | NR | 1.66 ± 0.92 |
| 3OC8 | 59.1 ± 7.5 | — | 33.2 ± 0.2 | NR | 6.77 ± 1.15 | 0.49 ± 0.11 | 5.47 ± 0.27 |
| 3OHC8 | — | — | — | 6.25 ± 1.33 | 0.36 ± 0.11 | — | 21.2 ± 6.6 |
| C10 | NR | — | 1.13 ± 0.06 | 1.49 ± 0.27 | 0.44 ± 0.13 | 42.0 ± 7.6 | — |
| 3OC10 | NR | — | NR | NR | 42.6 ± 21.8 nM | 4.00 ± 0.51 | — |
| 3OHC10 | NR | — | NR | NR | 15.0 ± 5.3 nM | — | NR |
| C12 | NR | — | NR | 11.0 ± 5.1 | 3.09 ± 0.44 | — | — |
| 3OC12 | 0.59 ± 0.13 | — | 1.90 ± 0.27 | 4.72 ± 2.23 | 0.40 ± 0.23 | 7.1 ± 2.0 | — |
| 3OHC12 | 48.4 ± 13.2 | — | 23.9 ± 9.2 | NR | NR | — | — |
| C14 | NR | — | — | NR | 0.47 ± 0.25 | — | — |
| 3OC14 | 2.35 ± 0.66 | — | NR | — | 13.9 ± 5.7 | — | — |
| 3OHC14 | 2.66 ± 0.68 | — | NR | — | 1.88 ± 0.78 | — | — |
| C16 | — | — | — | NR | — | — | — |
| 3OC16 | 7.05 ±  1.88 | — | — | — | 5.60 ± 0.13 | — | — |

^a^Cognate signals are shaded green. Values are mean ± SEM of n ≥ 3 independent experiments. — = does not activate. NR = not resolved.
