# Supplementary material for: Quorum Sensing Signal Selectivity and the Potential for Interspecies Cross Talk
Source: mBio. 2019 Mar 5;10(2):e00146-19. doi: 10.1128/mBio.00146-19 (PMC6401477; doi:10.1128/mBio.00146-19)
Supplement: TABLE S4 [file mBio.00146-19-st004.docx]

**Table S4.** EC_50_ values (µM unless indicated) for each indicated AHL for receptors expressed in *E. coli*^a^

| AHL | LasR | RhlR | QscR | BtaR1 | BtaR2 |
| --- | --- | --- | --- | --- | --- |
| C4 | — | 122 ± 17 | — | NR | — |
| 3OHC4 | — | — | — | NR | 25.0 ± 2.6 |
| C6 | — | 134 ± 26 | NR | NR | — |
| 3OC6 | — | NR | NR | — | — |
| 3OHC6 | — | — | — | — | NR |
| C8 | NR | NR | 1.90 ± 0.27 | 10.5 ± 3.6 nM | NR |
| 3OC8 | 17.4 ± 6.5 | NR | 0.12 ± 0.10 | 1.10 ± 0.29 | 12.4 ± 1.7 |
| 3OHC8 | NR | — | 5.82 ± 1.49 | 0.37 ± 0.14 | 77.8 ± 18.7 nM |
| C10 | 8.67 ± 1.49 | — | 3.11 ± 0.92 nM | 0.12 ± 0.08 | 1.68 ± 1.17 |
| 3OC10 | 0.21 ± 0.06 | — | 0.26 ± 0.16 | 2.83 ± 1.61 | 0.17 ± 0.07 |
| 3OHC10 | 7.91 ± 0.45 | — | 29.9 ± 13.4 nM | 1.82 ± 0.07 | 60.6 ± 16.0 nM |
| C12 | 0.66 ± 0.07 | — | 34.9 ± 20.8 nM | 1.90 ± 1.02 | 3.90 ± 0.91 |
| 3OC12 | 12.9 ± 3.6 nM | — | 53.4 ± 11.3 nM | 3.86 ± 0.91 | 0.29 ± 0.11 |
| 3OHC12 | 1.23 ± 0.86 | — | 0.11 ± 0.05 | NR | 91.5 ± 25.2 nM |
| C14 | 0.36 ± 0.13 | — | 89.6 ± 20.1 nM | 0.97 ± 0.35 | 4.29 ± 1.37 |
| 3OC14 | 12.5 ± 4.5 nM | — | 48.8 ± 21.3 nM | 1.26 ± 0.25 | 0.69 ± 0.26 |
| 3OHC14 | 0.20 ± 0.02 | — | 0.15 ± 0.04 | 2.23 ± 0.73 | 0.16 ± 0.03 |
| C16 | NR | — | — | NR | — |
| 3OC16 | 0.13 ± 0.03 | — | 0.17 ± 0.07 | 1.37 ± 0.27 | 0.31 ± 0.12 |

^a^Cognate signals are shaded green. Values are mean ± SEM of n ≥ 3 independent experiments. — = does not activate, NR = not resolved.
